# A warmer and drier climate in the northern sagebrush biome does not promote cheatgrass invasion or change its response to fire

**DOI:** 10.1007/s00442-017-3976-3

**Published:** 2017-10-16

**Authors:** Christian D. Larson, Erik A. Lehnhoff, Lisa J. Rew

**Affiliations:** 1 Weed and Invasive Plant Ecology and Management Group, Land Resources and Environmental Science Department, Montana State University, Bozeman, MT 59717 USA; 2 Entomology, Plant Pathology and Weed Science, New Mexico State University, Las Cruces, NM 88003 USA

**Keywords:** *Bromus tectorum*, Climate change, *Pseudoroegneria spicata*, Fire-feedback, Sagebrush biome

## Abstract

Dryland shrub communities have been degraded by a range of disturbances and now face additional stress from global climate change. The spring/summer growing season of the North American sagebrush biome is projected to become warmer and drier, which is expected to facilitate the expansion of the invasive annual grass *Bromus tectorum* (cheatgrass) and alter its response to fire in the northern extent of the biome. We tested these predictions with a factorial experiment with two levels of burning (spring burn and none) and three climate treatments (warming, warming + drying, and control) that was repeated over 3 years in a Montana sagebrush steppe. We expected the climate treatments to make *B. tectoru*m more competitive with the native perennial grass community, especially *Pseudoroegneria spicata*, and alter its response to fire. Experimental warming and warming + drying reduced *B. tectorum* cover, biomass, and fecundity, but there was no response to fire except for fecundity, which increased; the native grass community was the most significant factor that affected *B. tectorum* metrics. The experimental climate treatments also negatively affected *P. spicata,* total native grass cover, and community biodiversity, while fire negatively affected total native grass cover, particularly when climate conditions were warmer and drier. Our short-term results indicate that without sufficient antecedent moisture and a significant disruption to the native perennial grass community, a change in climate to a warmer and drier spring/summer growing season in the northern sagebrush biome will not facilitate *B. tectorum* invasion or alter its response to fire.

**Electronic supplementary material:**

The online version of this article (doi:10.1007/s00442-017-3976-3) contains supplementary material, which is available to authorized users.

## Introduction

The sagebrush biome covers more than 43 million hectares and is one of the largest ecosystems in North America (Rowland et al. 2010). It provides productive rangelands (Rowland et al. 2010), acts as an important carbon sink (Gilmanov et al. 2006), and fosters biodiverse native communities that provide habitat for threatened species (Miller et al. 2011). This region has an extensive history of disturbance (grazing, fire, development) (Knapp 1996; Rowland et al. 2010), which continues today. Understanding the effects of these disturbances in a changing climate is important for maintaining ecosystem diversity and productivity. One of the most significant results of disturbance within this region has been its role in the spread of non-native invasive plant species, which negatively impact the region’s biodiversity and productivity (Rowland et al. 2010).

The non-native invasive plant species that has had the most negative impact and poses the greatest threat to sagebrush ecosystems is the winter annual grass *Bromus tectorum* (Suring et al. 2005). *Bromus tectorum*, accidently introduced in the 1880s, was widespread throughout western North America by the 1920s (Mack 1981), and is currently naturalized throughout much of North America (Morrow and Stahlman 1984). As a winter annual, *B. tectorum* can germinate in the fall, winter, or spring (Morrow and Stahlman 1984), which affords it a competitive edge over native seedlings (Morrow and Stahlman 1984). However, this early germination and its subsequent growth requires suitable climate conditions, most importantly ample winter/spring soil moisture availability (Bradford and Lauenroth 2006; Bradley et al. 2016).

*Bromus tectorum’s* invasion has been closely tied with anthropogenic disturbance; grazing facilitated its dispersal and establishment, and a positive feedback with fire has led to its ecological dominance in some areas (Mack 1981; Knapp 1996; Taylor et al. 2014). This dominance has largely been constrained to the Great Basin, Columbia Plains, and Colorado Plateau regions (Mack 1981; Knapp 1996; Bradley 2009; Brummer et al. 2016; Downs et al. 2016). Research into the mechanisms behind this dominance and what has constrained it to these regions has demonstrated the importance of the dynamic between the native grass community, disturbance, and climate (Chambers et al. 2014a, b).

Ecosystem resilience is an ecosystem’s capacity to regain fundamental structure, processes, and functioning after stresses or disturbances (Chambers et al. 2014a), while an ecosystem’s resistance to invasion is a function of the attributes and ecological processes that limit an invading species (Chambers et al. 2014a). Robust, undisturbed native perennial grass communities are resistant to *B. tectorum* invasion and limit its dominance (Chambers et al. 2007; Brummer et al. 2016), while disturbance (especially fire and grazing) of these communities increases the risk of *B. tectorum* invasion (Mack 1981). This makes the resilience of native ecosystems to disturbance important for how resistant the community is to *B. tectorum* invasion (Chambers et al. 2014a). Temperature and precipitation regimes affect sagebrush ecosystem resilience to fire (Chambers et al. 2014a); warmer and drier ecosystems are less resilient to fire than cooler and wetter ecosystems (Chambers et al. 2007, 2014a, b). The lower resilience of warmer and drier ecosystems has made them more susceptible to *B. tectorum* invasion following disturbance (Chambers et al. 2007, 2014a, b; Taylor et al. 2014). Consistent with these studies, the combination of warm and dry summer climate conditions is a key factor defining *B. tectorum* invasiveness throughout the sagebrush biome (Bansal and Sheley 2016; Brummer et al. 2016). Similarly, areas where the *B. tectorum*-fire cycle has been found are warmer and receive less summer precipitation than those areas where *B. tectorum* has yet to demonstrate a positive feedback with fire (Taylor et al. 2014).

Climate models for the northern sagebrush biome project temperatures to increase by 2–4 °C by 2100 and, while there is a lot or variation in the model projections for precipitation, summer precipitation is projected to decrease (Mote and Salathé 2010; Pederson et al. 2010; Polley et al. 2013) and soils to become drier in the summer resulting in greater plant water stress (Bradley et al. 2016). These changes will potentially make ecosystems more climatically similar to the rest of the biome and, thus, less resilient to disturbance and resistant to *B. tectorum* invasion. Accordingly, given ample late winter/early spring soil moisture availability, climate envelope models have projected that as summer temperatures warm and spring/summer precipitation and soil moisture decrease there will be an expansion of both *B. tectorum* dominance and its positive feedback with fire at higher elevations and latitudes (Bradley 2009; Taylor et al. 2014; Bradley et al. 2016). Recent *B. tectorum* climate manipulation studies in Utah and southern Wyoming have demonstrated that experimentally increased temperatures positively affect *B. tectorum* (Zelikova et al. 2013; Compagnoni and Adler 2014a, b; Blumenthal et al. 2016), and *B. tectorum* has expanded along its high elevation range margin in Colorado (Bromberg et al. 2011). Similarly, experimentally reduced precipitation of an invaded Colorado mixed prairie positively affected *B. tectorum* seed production and cover (Prevéy and Seastedt 2015). While a study that employed a prescribed burn in the fall found the treatment to negatively affect overwinter *B. tectorum* seedling survival, it also demonstrated that burning increased *B. tectorum* biomass and fecundity (Chambers et al. 2007). Finally, both observational and experimental fire studies, with sites in the Great Basin and Columbia Basin regions, have found that ecosystem resilience to fire is lowest in warm and dry sites and greatest in cool and moist sites, resulting in greater *B. tectorum* invasion at the warm and dry sites after fire (Chambers et al. 2007, 2014b; Dodson and Root 2016), especially if the perennial grasses and forbs have been reduced.

*Bromus tectorum* is naturalized in Montana, however, in this relatively cold and wet northern region of the sagebrush biome there have not been any documented cases of *B. tectorum* dominating natural ecosystems by forming dense monocultures, nor any cases of the positive *B. tectorum*-fire cycle (Taylor et al. 2014). A change in climate may not only facilitate the spread of *B. tectorum* within this region but could also effect a change from its current subordinate community role to what has been referred to as a ‘transformer’, initiating a *B. tectorum*-fire cycle (Richardson et al. 2000; Hellmann et al. 2008; Taylor et al. 2014). At the time of this study, small-scale field experiments have addressed the possibility of a *B. tectorum* range shift by elevating temperatures (Zelikova et al. 2013; Compagnoni and Adler 2014a, b; Blumenthal et al. 2016) and altering precipitation patterns (Prevéy and Seastedt 2015). However, no studies have experimentally increased the temperature and decreased the precipitation of *B. tectorum* communities. Likewise, no studies have addressed the potential for climate change to induce a *B. tectorum* community role change by altering the climate factors of recently burned *B. tectorum* communities, particularly at a northerly latitude where it is limited by cold and relatively dry winters and wet summers.

The goal of this study was to assess the responses of *B. tectorum, Pseudoroegneria spicata,* and the native plant community in a northern sagebrush steppe site to experimentally increased growing season temperatures and decreased growing season precipitation, in addition to a spring burn. The questions of our study were: (1) How will *B. tectorum’s* and *P. spicata’s* abundance (cover and biomass) and fecundity, as well as native plant community cover, respond to experimental growing season warming? (2) How will these same response variables respond to experimental growing season warming + drying? We expected to find a negative relationship between the native grass community and *B. tectorum*. Thus, our next question was: (3) How will the interactions between *B. tectorum* and *P. spicata,* and between *B. tectorum* and the native grass community be affected by the two climate treatments? Similarly, (4) how will *B. tectorum*’*s* impact on community biodiversity be altered by the climate treatments? Our final question: (5) What effect will a spring prescribed burn have on the aforementioned *B. tectorum*, *P. spicata*, and native community response variables under experimentally warmed and dried conditions?

## Methods

### Study site

The study site was a sagebrush steppe rangeland located 56 km west of Bozeman, MT, at the Montana State University Red Bluff Agricultural Research Station in Norris, MT, USA (5049898.184N, 451464.866E (UTM)) at an elevation of 1600 m. Site vegetation was dominated by the native species *Ericameria nauseosa*, *Artemisia tridentata*, and *Artemisia frigida* in the shrub layer, and *P. spicata, Stipa comata*, *Lupinus argenteus*, and *Artemisia ludviciana* in the herbaceous layer. *Bromus tectorum* was the most abundant non-native species. Other non-native species included: *Alyssum desertorum*, *Sisymbrium altissimum*, and *Tragopogon dubious* (using nomenclature of Lesica et al. (2012)). The soils of the Red Bluff site were part of the Nuley-rock outcrop complex; sandy loam (0–10 cm), sandy clay loam (10–28 cm), gravelly sandy loam (28–61 cm) (http://websoilsurvey.nrcs.usda.gov/app/websoilsurvey.aspx).

Temperatures during our study were similar but warmer than the historical averages of the Norris climate station, 16 km south of the Red Bluff research station (Online Resource 1; NCEI 2016). Over the last 30 years (1984–2013), the coldest quarter (January–March) has averaged 0.85 °C, while the warmest quarter (July–September) has averaged 19.35 °C. During the same 30-year period (1984–2013), April–June have been the wettest months receiving an average of 46% of the annual precipitation, and January–March have been the driest months receiving an average of 12% of the annual precipitation (Online Resource 1; NCEI 2016).

### Experimental design

The research questions were addressed using two burn treatments (burned and unburned), and three different climate treatments (control, warming, warming + drying). Given the relative importance of summer temperature in defining *B. tectorum* distribution and invasion compared to winter temperatures (Bradley 2009; Taylor 2014; Bradley et al. 2016; Brummer et al. 2016) and the climate predictions in our area that project increased summer temperatures (Mote and Salathé 2010; Pederson et al. 2010; Polley et al. 2013) our warming treatments were implemented between April and October for 3 years (2014, 2015, 2016). At the beginning of each growing season, new 2 m^2^ plots were randomly located within a 30 m × 60 m grid, which had a uniform slope and aspect, and natural densities of *B. tectorum* and *P. spicata*. The six climate-burn treatments were replicated ten times in 2014, after which, a power test was conducted and the number of replicates was reduced to six for 2015 and 2016.

In accordance with local fire restrictions, at the beginning of each year the 2 m^2^ plots implemented that year were burned in early April. Consistent with prescribed burning methods (Sirois 1993; Kral et al. 2015) a propane torch was used (Red Dragon model VT21/2 vapor propane torch by Flame Engineering Corporation, La Crosse, Kansas) in conjunction with a temporary fire block created out of aluminum flashing material (Jones et al. 2015). The climate manipulation structures (see below) were established in April immediately after the burn treatment. Delmhorst soil moisture measuring systems (model KS-D1) were installed in the center of each plot at a depth of 10 cm (Aho and Weaver 2008) and were read regularly. Temperatures were recorded (3 hourly) using Maxim Integrated thermochron iButton devices (DS1921G; − 40 °C: − 85 °C) that were deployed in each plot on the north side of a stake 20 cm above the soil surface.

### Climate manipulation designs

*Open top chambers*. Open top chambers (OTC) were used to increase the temperature of the warming and warming + drying treatments, following the cone chamber design (Molau and Molgaard 1996; Marion et al. 1997). The OTC design was intended to increase mean temperatures by 2 °C. They were constructed out of Sun-Lite HP (1 mm thick) (Solar Components Corporation, Manchester, New Hampshire) fiberglass material that has relatively high solar transmittance in the visible wavelengths (86%) and a low transmittance in the infra-red range (< 5%). The chambers had basal diameters of 1.5 m and top openings with diameters of 1.0 m. The chambers were 0.4 m tall with a 60° incline (Marion et al. 1997).

*Rainout shelters*. Rainout shelters following the design specified in Yahdjian and Sala (2002) were placed over OTCs to decrease the precipitation for the warming + drying treatment by 55%. They were constructed with wooden frames supporting gutters made of corrugated clear polycarbonate material (Suntuf) to remove precipitation from the plots. Suntuf clear polycarbonate was used because of its high light transmittance of 90%, and its toughness and flexibility to withstand high winds and inclement weather. The gutters extended 0.10 m beyond the OTC on the high side and 0.20 m on the low side to maximize interception and to prevent capillary action of soil water from inadvertently watering the vegetation within the plot. To maximize their effectiveness, the rainout shelters were oriented southwest towards the prevailing winds.

### Sampling methods

Plots were only sampled throughout the growing season in which they were implemented. To account for edge effects, sampling took place within a smaller 0.75 m^2^ area centered in each of the treated 2 m^2^ plots. Total aerial cover (%), density of reproductive tillers, aboveground biomass, the number of seeds produced plot^−1^, and individual fecundity (seeds produced stem^−1^) were assessed for both target species (*B. tectorum* and *P. spicata*). Cover was assessed visually by the same two observers, who calibrated their estimates at the beginning of each sampling day. Density of reproductive tillers was assessed at the plot level throughout the growing season. In all years, destructive sampling (clipping) of aboveground *B. tectorum* biomass occurred on June 30th, while *P. spicata* biomass sampling took place on July 14th. For both species, individual fecundity was assessed on a subsample of 10 stems for each plot at seed maturity by counting all filled seeds. In addition, each year a community assessment was taken on June 30th, during which the cover was assessed for all species individually.

### Statistical analysis

Temperature and soil moisture data were analyzed using linear mixed-effects models, treating plot and time (Julian day for each year) as random effects and the climate and burned treatments as fixed effects. As we were interested in plant available soil water, we constrained our analysis to soil moisture above the permanent wilting point (− 15 bars).

Linear mixed-effects models were also used to assess the effects of climate, the burned treatment, and other explanatory variables on the *B. tectorum* response variables (cover, aboveground biomass, reproductive tiller density, seeds produced plot^−1^, and individual fecundity). The experimental treatments (climate manipulation and burn), along with native grass cover and forb cover were treated as fixed effects, while year was treated as a random effect. Initially, the relative importance of the different predictor variables (climate and burn treatments, the interaction between these two experimental treatments, forb and native grass cover) on *B. tectorum*’s cover was assessed using the difference in Akaike Information Criterion (AIC) values. A difference of two in AIC values between models was used as the threshold indicating a better fit model. All further analysis of *B. tectorum* response variables compared nested models, beginning with the full model that included all explanatory variables that were fully crossed. Variables and interactions that did not explain a considerable amount of variability within the data were removed until the best and most parsimonious model that included the experimental climate and burn treatments was obtained.

Linear mixed-effects models were also used to assess the effects of the experimental treatments and *B. tectorum* cover on the *P. spicata* response variables (cover, aboveground biomass, reproductive tiller density, seeds produced plot^−1^, and individual fecundity), and native grass cover and community diversity (Simpson’s and Shannon’s diversity indices). The same approach as described above was used to discern between models. Species richness data (total and native) were analyzed using generalized linear mixed-effects models with a Poisson distribution. To avoid bias, because we used *B. tectorum* cover as a predictor variable, it was excluded from all biodiversity calculations.

To satisfy assumptions of normality and constant variance, *B. tectorum* and *P. spicata* variables were naturally log transformed. These assumptions were assessed visually and using the Breusch–Pagan test. Significant differences between predictor variables and response variables at the *P* < 0.05 level were calculated from T statistics based on Satterthwaite’s approximations of degrees of freedom for linear mixed-effects models (Kuznetsova et al. 2014). Data were analyzed using the lme4 package (Bates et al. 2011) and the lmerTest package (Kuznetsova et al. 2014) in the statistical analysis program R (R Development Core team 2015).

## Results

The open top chambers significantly increased the mean and maximum temperatures of the warming, and warming + drying treatments, while the warming + drying treatment also increased minimum temperatures (Table 1; Online Resources 2–4). The burned treatment did not affect temperatures. The mean soil water potential for each of the 3 years (2014, 2015, 2016) demonstrated that the rainout shelters reduced water potential in all years (Online Resources 5–7). The burned treatment did not affect soil moisture. The shelters were designed to reduce precipitation by 55%. We did not quantify the exact reduction in precipitation but assessed soil moisture, as it is more directly relevant to plant growth.

**Table 1:** Average mean, minimum, and maximum temperatures within the ambient, warming, and warming + drying climate treatments recorded at Red Bluff Research Station (2014–2016) imposed from April–October

Year	Treatment	Mean (°C)	Minimum (°C)	Maximum (°C)
April				
2015	Ambient	7.19^a^	− 2.78^a^	19.77^a^
	Warming	8.80^b^	− 2.77^a^	24.23^b^
	Warming + drying	9.10^b^	− 1.75^b^	23.51^b^
2016	Ambient	9.24^a^	1.30^a^	20.76^a^
	Warming	10.70^b^	1.03^b^	25.34^b^
	Warming + drying	11.09^b^	1.66^c^	24.96^b^
May–July				
2014	Ambient	17.00^a^	5.86^a^	29.77^a^
	Warming	18.40^b^	5.85^a^	34.00^b^
	Warming + drying	18.68^b^	6.53^b^	32.57^c^
2015	Ambient	18.08^a^	7.76^a^	31.62^a^
	Warming	19.96^b^	7.80^a^	36.25^b^
	Warming + drying	20.15^b^	8.74^b^	35.05^b^
2016	Ambient	18.44^a^	6.64^a^	32.28^a^
	Warming	20.16^b^	6.31^b^	37.08^b^
	Warming + drying	20.58^b^	7.29^c^	36.36^b^

Data shown here represents the growing season (April–July) effects of the treatments. 2014 April temperature data were not available. Yearly data were analyzed separately and superscripts indicate statistical significance (*P* < 0.05)

**Fig. 1:**
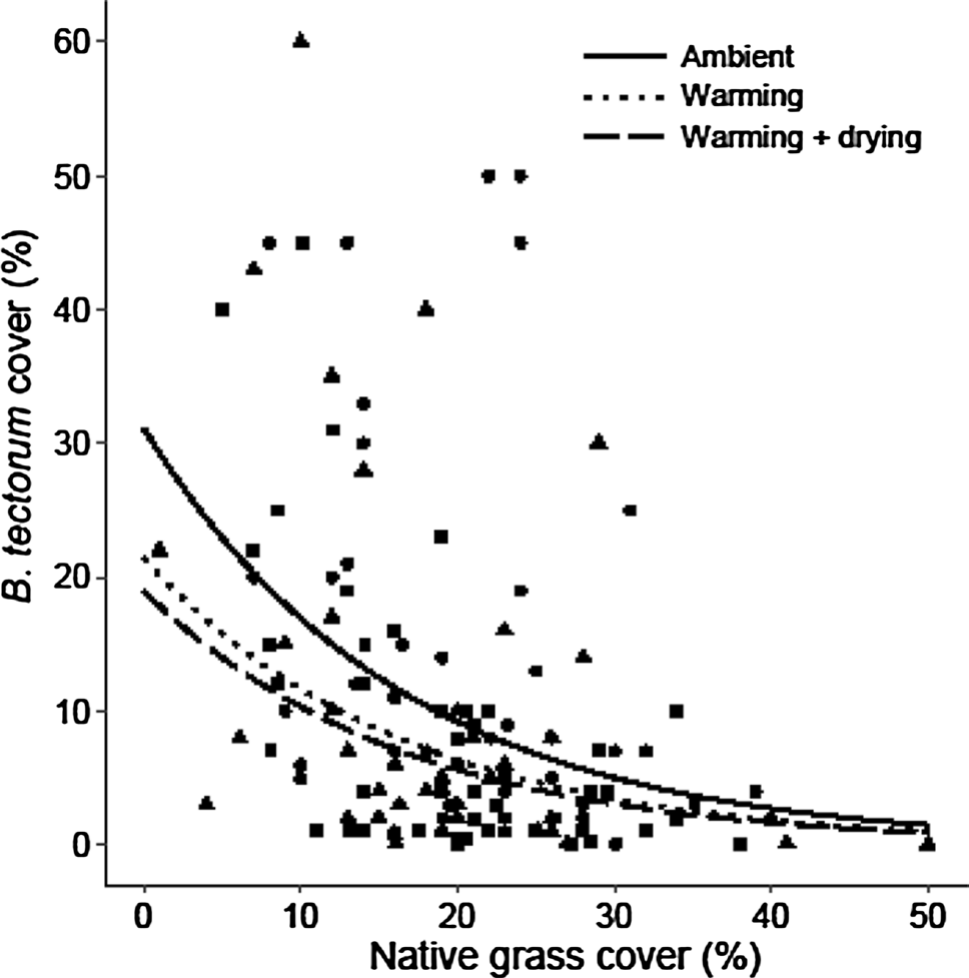
*Bromus tectorum* cover response to native grass cover within ambient (circles, solid line), warming (triangles, dotted line), and warming + drying (squares, dashed line) climate treatment plots (0.75 m^2^). Results of a linear mixed-effects model demonstrated that *B. tectorum* cover was negatively affected by native grass cover (*n* = 132, *P* < 0.001), warming (*n* = 44, *P* = 0.035), and warming + drying (*n* = 44, *P* = 0.007)

Total community native grass cover was the most important variable explaining the variation in *B. tectorum* cover (%) followed by the climate treatments, while other variables accounted for little of the variation within the data (Table 2). As such, the best cover model included both experimental treatments (climate and burn) and total native grass community cover (%). As expected, the results of the model demonstrated a negative relationship between *B. tectorum* cover and total native grass cover (*P* < 0.001). However, contrary to our expectations, the warming treatment negatively affected *B. tectorum* cover (*P* = 0.035; Table 3; Fig. 1). *B. tectorum* cover also responded negatively to the warming + drying treatment (*P* = 0.007, respectively; Table 3; Fig. 1). The burned treatment did not affect *B. tectorum* cover (*P* = 0.105; Table 3).

**Table 2:** Akaike Information Criterion (AIC) table testing the effects of vegetation cover and the climate and burned treatments on *Bromus tectorum* cover

Model	Tested factor	AIC	Δ AIC
•Full model	NA	338.12	0
•Full-forb cover	Forb cover	336.84	− 1.28
•Full-native grass cover	Native grass cover	377.29	39.17
*Climate + veg	Burn treatment	337.40	− 0.72
*Burn + veg	Climate treatment	341.23	3.11
*Climate + burn + veg	Climate × burn interaction	336.54	− 1.58

Delta (Δ) AIC indicates the relative importance of the tested explanatory variable (higher delta values indicate those factors that were most important)

•Full model = lmer (ln(*B. tectorum* cover) ~ (climate x burn status) + native grass + forb + (1 year)

*Veg = Native grass cover + forb cover

**Table 3:** Results of the best linear mixed-effects models assessing *Bromus tectorum* responses to the burned, warming, warming + drying treatments, and native grass cover

Fixed effects							Random effects	
Response	Predictor	Est.	SE	*df*	*t* value	*P* (>)	Variance	
							Year	Residual
Cover (%)	Intercept	3.65	0.43	4.50	8.54	< 0.001	0.34 ± 0.59	0.70 ± 0.84
	Burned	− 0.25	0.15	120.98	− 1.63	0.105		
	Warming	**− 0.39**	**0.18**	**120.99**	**− 2.14**	**0.035**		
	Warming + drying	**− 0.50**	**0.18**	**120.97**	**− 2.77**	**0.007**		
	Native grass cover (%)	**− 0.06**	**0.01**	**121.21**	**− 7.00**	**< 0.001**		
Aboveground biomass (g)	Intercept	3.23	0.47	6.54	6.83	< 0.001	0.43 ± 0.66	0.82 ± 0.90
	Burned	− 0.16	0.17	125.01	− 0.95	0.345		
	Warming	− 0.35	0.20	125.02	− 1.77	0.080		
	Warming + drying	**− 0.45**	**0.19**	**125.00**	**− 2.32**	**0.022**		
	Native grass cover (%)	**− 0.05**	**0.01**	**125.23**	**− 5.33**	**<0.001**		
Reproductive density (tillers plot^−1^)	Intercept	6.80	0.60	7.51	11.54	< 0.001	0.61 ± 0.78	1.47 ± 1.21
	Burned	− 0.41	0.22	124.95	− 1.86	0.065		
	Warming	**− 0.69**	**0.26**	**124.97**	**− 2.62**	**0.010**		
	Warming + drying	**− 0.95**	**0.26**	**124.94**	**− 3.65**	**< 0.001**		
	Native grass cover (%)	**− 0.08**	**0.01**	**125.23**	**− 6.14**	**< 0.001**		
Seed production (seeds plot^−1^)	Intercept	8.21	0.89	5.58	9.26	< 0.001	1.67 ± 1.29	2.11 ± 1.45
	Burned	0.43	0.27	120.00	1.58	0.117		
	Warming	**− 0.77**	**0.32**	**120.00**	**− 2.36**	**0.020**		
	Warming + drying	**− 0.92**	**0.32**	**120.01**	**− 2.87**	**0.005**		
	Native grass cover (%)	**− 0.09**	**0.02**	**120.12**	**− 4.77**	**< 0.001**		
Individual fecundity (mean seeds stem^−1^)	Intercept	2.02	0.47	3.88	4.32	0.013	0.57 ± 0.76	0.26 ± 0.51
	Burned	**0.53**	**0.09**	**120.01**	**5.62**	**< 0.001**		
	Warming	− 0.12	0.11	120.02	− 1.08	0.282		
	Warming + drying	− 0.18	0.11	120.02	− 1.58	0.116		
	Native grass cover (%)	**− 0.01**	**0.01**	**120.06**	**− 2.14**	**0.034**		

Values in bold indicate statistically significant differences (*P* < 0.05). Response variables were assessed at the plot level (0.75 m^2^), except individual fecundity, which is mean seeds stem^−1^

**Fig. 2:**
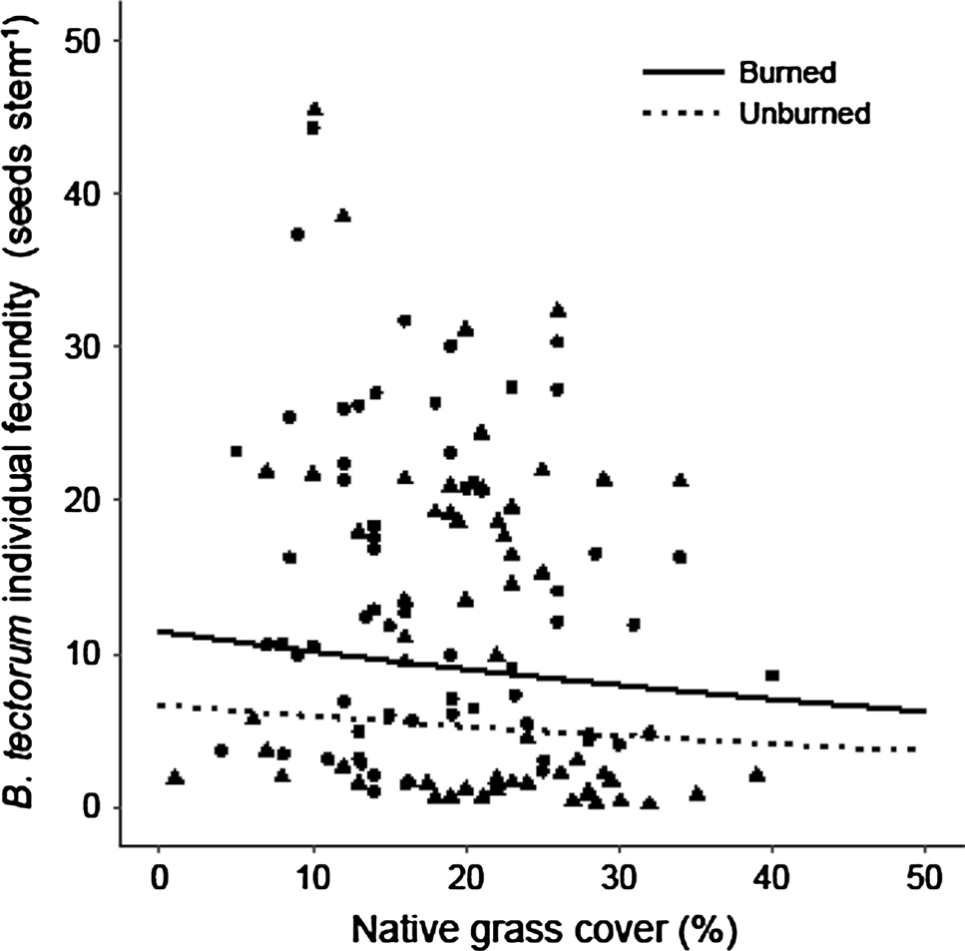
*Bromus tectorum* individual fecundity (seeds stem^−1^) response to the native grass cover within burned (circles, solid line), and unburned (triangles, dotted line) plots (0.75 m^2^). Results of a linear mixed-effects model demonstrated that *B. tectorum* fecundity was negatively affected by native grass cover (*n* = 132, *P* = 0.034) and positively affected by the burned treatment (*n* = 66, *P* < 0.001)

*Bromus tectorum* aboveground biomass (g) also demonstrated a negative relationship with total native grass community cover (*P* < 0.001) and to the warming + drying treatment (*P* = 0.022; Table 3). Neither the warming treatment nor the burned treatment affected *B. tectorum* biomass (*P* = 0.080 and *P* = 0.345, respectively; Table 3). *Bromus tectorum* reproductive density (tiller plot^−1^) and seed production (seeds plot^−1^) demonstrated negative relationships with the total native grass community cover (*P* < 0.001 and *P* < 0.001, respectively). They were also negatively affected by both the warming (*P* = 0.010 and *P* = 0.020, respectively) and the warming + drying climate treatments (*P* < 0.001 and *P* = 0.005, respectively; Table 3). However, they were not significantly affected by the burned treatment (*P* = 0.065 and *P* = 0.117, respectively; Table 3). Individual *B. tectorum* fecundity (seeds stem^−1^) was not affected by either climate treatment, but responded negatively to total community native grass cover (*P* = 0.034). Interestingly, *B. tectorum* individual fecundity responded positively to the burned treatment (*P* < 0.001; Fig. 2; Table 3).

**Fig. 3:**
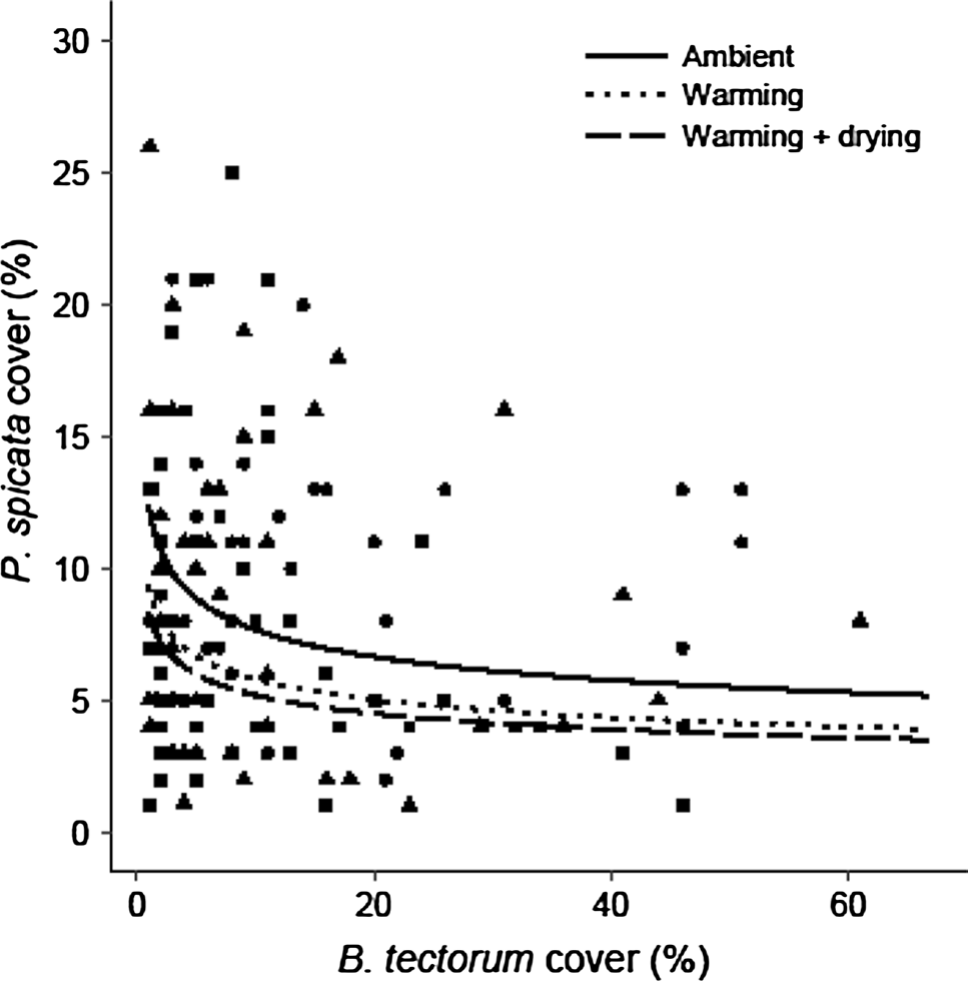
*Pseudoroegneria spicata* cover response to *B. tectorum* cover within the ambient (circles, solid line), warming (triangles, dotted line), and warming + drying (squares, dashed line) climate treatment plots (0.75 m^2^). Results of a linear mixed-effects model demonstrated *P. spicata* cover was lower in the presence of *B. tectorum* (*n* = 132, *P* = 0.001) and was negatively affected by the warming +drying climate treatment (*n* = 44, *P* = 0.001). *Pseudoroegneria spicata* cover was not affected by the warming treatment (*n* = 44, *P* = 0.061)

*Pseudoroegneria spicata* cover (%) (Fig. 3), aboveground biomass (g), reproductive density (tiller plot^−1^), and seed production (seeds plot^−1^) were lower in the presence of *B. tectorum* (*P* = 0.001, *P* = 0.012, *P* = 0.010, *P* = 0.017, respectively; Table 4). *Pseudoroegneria spicata* cover (Fig. 3) and biomass responded negatively to the warming + drying treatment (*P* = 0.010 and *P* = 0.005). However, neither metric was affected by the warming or the burned treatments (Table 4). Both *P. spicata* reproductive density and seed production were negatively affected by the warming treatment (*P* = 0.020 and *P* = 0.043, respectively) and the warming + drying treatment (*P* < 0.001 and *P* < 0.001, respectively) but not the burned treatment (Table 4). *Pseudoroegneria spicata* individual fecundity (seeds stem^−1^) was not affected by any of the experimental treatments, or *B. tectorum* cover (Table 4).

**Table 4:** Results of the best linear mixed-effects models assessing *Pseudoroegneria spicata* responses to the burned, warming, warming + drying treatments, and *Bromus tectorum* cover

Fixed effects							Random effects	
Response	Predictor	Est.	SE	*df*	*t* value	*P* (>)	Variance	
							Year	Residual
Cover (%)	Intercept	2.55	0.28	4.81	9.20	< 0.001	0.13 ± 0.37	0.47 ± 0.68
	Burned	− 0.08	0.12	121.03	− 0.66	0.511		
	Warming	− 0.28	0.15	121.03	− 1.89	0.061		
	Warming + drying	**− 0.39**	**0.15**	**121.10**	**− 2.61**	**0.010**		
	*B. tectorum* cover (%)	**− 0.21**	**0.06**	**122.96**	**− 3.31**	**0.001**		
Aboveground biomass (g)	Intercept	1.82	0.40	2.99	4.57	0.020	0.38 ± 0.61	0.47 ± 0.68
	Burned	− 0.14	0.12	121.04	− 1.14	0.256		
	Warming	− 0.26	0.15	121.04	− 1.71	0.090		
	Warming + drying	**− 0.53**	**0.15**	**121.07**	**− 3.56**	**0.005**		
	*B. tectorum* cover (%)	**− 0.16**	**0.06**	**122.36**	**− 2.54**	**0.012**		
Reproductive density (tillers plot^−1^)	Intercept	3.30	0.54	5.37	6.10	0.001	0.63 ± 0.79	1.21 ± 1.10
	Burned	− 0.19	0.20	125.05	− 0.99	0.326		
	Warming	**− 0.57**	**0.24**	**125.05**	**− 2.35**	**0.020**		
	Warming + drying	**− 1.03**	**0.24**	**125.09**	**− 4.30**	**< 0.001**		
	*B. tectorum* cover (%)	**− 0.26**	**0.10**	**127.11**	**− 2.60**	**0.010**		
Seed production (seeds plot^−1^)	Intercept	6.10	0.93	3.75	6.53	0.004	1.82 ± 1.35	3.85 ± 1.96
	Burned	0.31	0.35	121.06	− 0.89	0.374		
	Warming	**− 0.88**	**0.43**	**121.07**	**− 2.05**	**0.043**		
	Warming + drying	**− 1.76**	**0.43**	**121.11**	**− 4.12**	**< 0.001**		
	*B. tectorum* cover (%)	**− 0.43**	**0.18**	**122.85**	**− 2.41**	**0.017**		
Individual fecundity (mean seeds stem^−1^)	Intercept	2.75	0.10	26.61	27.98	< 0.001	0.005 ± 0.07	0.11 ± 0.33
	Burned	− 0.08	0.06	107.37	− 1.29	0.200		
	Warming	− 0.01	0.08	107.66	− 0.19	0.848		
	Warming + drying	− 0.09	0.08	107.52	− 1.17	0.244		
	*B. tectorum* cover (%)	− 0.02	0.03	91.96	− 0.67	0.504		

Values in bold indicate statistically significant differences (*P* < 0.05). Response variables were assessed at the plot level (0.75 m^2^), except individual fecundity, which is mean seeds stem^−1^

**Fig. 4:**
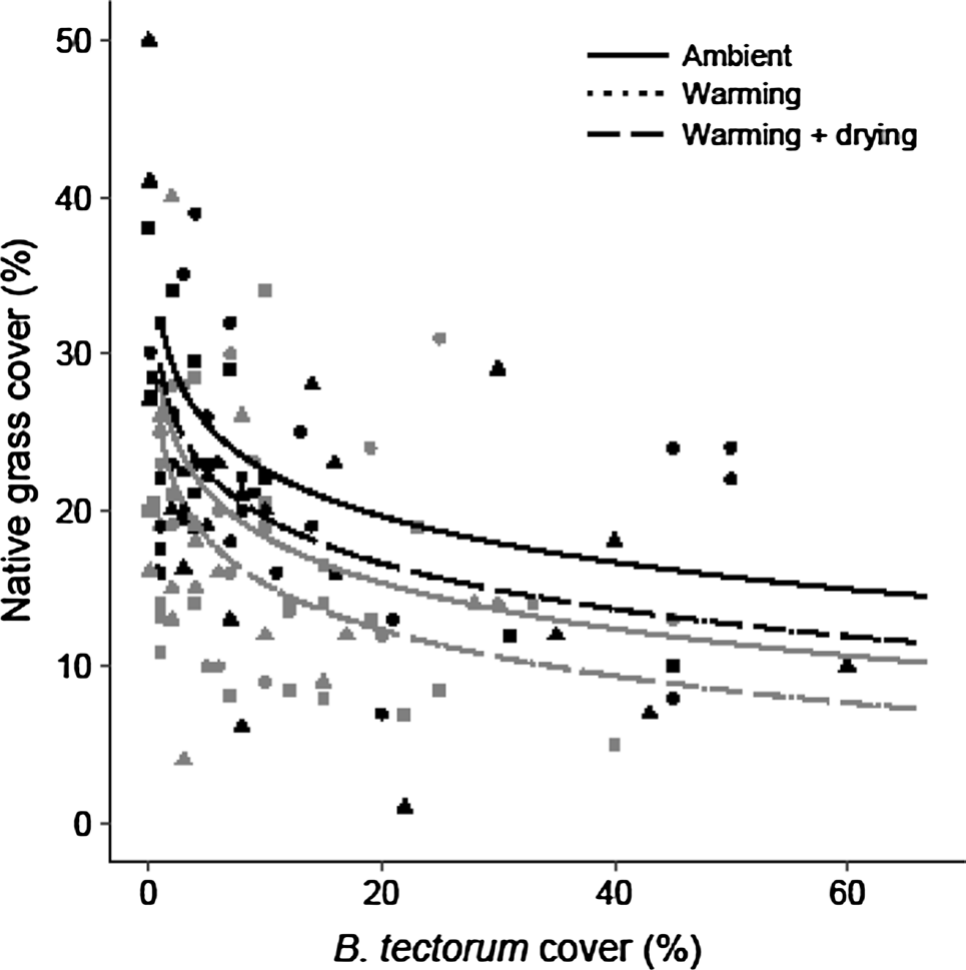
Native grass cover in the ambient (circles, solid line), warming (triangles, dotted line), and warming + drying (squares, dashed line) climate treatment plots (0.75 m^2^) for unburned (black) and burned (gray) fire treatments. Results of a linear mixed-effects model demonstrated that native grass cover was lower in the presence of *B. tectorum* (*n* = 132, *P* < 0.001) and was negatively affected by the warming and warming + drying climate treatments (*n* = 44, *P* = 0.044, 0.045, respectively), as well as the burned treatment (*n* = 66, *P* < 0.001). The native grass cover trend lines for both climate treatments occlude each other for the burn and unburned treatments

Analysis of the total native grass community cover demonstrated that it was reduced in the presence of *B. tectorum* (*P* < 0.001), and negatively affected by the warming, and warming + drying climate treatments (*P* = 0.044 and *P* = 0.045, respectively), as well as the burned treatment (*P* < 0.001; Fig. 4). Total and native species richness, as well as Simpson’s and Shannon’s diversity indices were negatively affected by *B. tectorum* cover (*P* < 0.001, *P* < 0.001, *P* = 0.030, *P* < 0.001, respectively). Species richness, both total and native, and the Simpson’s diversity index were not affected by either climate treatment or the burned treatment. However, the warming + drying treatment negatively affected the Shannon’s diversity index (*P* = 0.040).

## Discussion

### Responses to elevated temperatures

One of the most documented and significant ecological effects of increasing temperatures associated with global climate change is the impact it has had on species range distributions; many species range have shifted up in elevation and poleward in latitude (McCarty 2001; Walther et al. 2002; Parmesan 2006; Lenoir and Svenning 2014). A meta-analysis of studies that experimentally manipulated climate factors found that experimental warming stimulated photosynthesis and plant growth (Wu et al. 2011). Similarly, field experiments have demonstrated that when there is sufficient water available for growth *B. tectorum* has responded positively to experimentally increased temperatures (Zelikova et al. 2013; Compagnoni and Adler 2014a, b; Blumenthal et al. 2016). Therefore, contrary to our expectations, we found *B. tectorum*, *P. spicata*, and total native grass cover all responded negatively to experimental warming.

Our warming treatment (+ ~ 1.5 °C) was similar to previous *B. tectorum* warming studies, which warmed between 1.5 and 7 °C (Zelikova et al. 2013; Compagnoni and Adler 2014a, b; Blumenthal et al. 2016). However, the majority of these studies warmed continuously throughout the winter, as well as during the growing season. It is unclear if warming during the winter at our site would have elicited a positive response by *B. tectorum*. Cold temperatures decrease *B. tectorum* germination (Roundy et al. 2007), and low soil temperatures constrain its establishment, growth, and reproduction (Chambers et al. 2007; Bradley et al. 2016). However, over-winter warming would have also resulted in a reduced snowpack, which, in previous studies at cold locations, has resulted in exposing *B. tectorum* to more extreme cold events and decreased over winter survival (Griffith and Loik 2010). In addition, a recent observational study conducted at sites with climates similar to ours (cold and dry winters, warm and wet summers) found that overall annual brome grass abundance (including *B. tectorum*) was lowest when the winter temperatures were warmer than the historical average (Ashton et al. 2016), presumably due to overwinter mortality. Therefore, the more likely explanation as to why our *B. tectorum*, and *P. spicata,* responded negatively to our warming treatments is that our site receives less late winter/early spring precipitation than studies conducted to the south, and our warming increased evapotranspiration and heightened plant water stress during the spring–summer growing season.

### Responses to seasonality of precipitation and soil moisture availability

Precipitation patterns, specifically timing of precipitation, strongly influences *B. tectorum* establishment, growth, and reproduction (Bradford and Lauenroth 2006; Prevéy and Seastedt 2015; Bradley et al. 2016). Two Colorado field studies demonstrated that when winter precipitation in the form of rain was increased *B. tectorum* responded positively, and when it was reduced, *B. tectorum* responded negatively (Prevéy and Seastedt 2014, 2015). On sites that averaged 85, 128, and 122 mm of total precipitation between January and March (wrcc.dri.edu/summary), Compagnoni and Adler (2014a) found *B*. *tectorum* fecundity and survival responded positively to experimentally increased temperatures. Contrasting with this, our site only received 47 and 79 mm of total precipitation between January and March in 2015 and 2016, respectively, which is normal for our area. In these years, the *B. tectorum* response was significantly negative. In 2014, when our site received a similar amount of January–March precipitation (128 mm) to the more southerly studies, *B. tectorum* responded neutrally to the warming treatment. This same trend was reported in another study: Zelikova et al. (2013) found that *B. tectorum* responded positively to experimental warming when there was ample (110 mm) precipitation between January and March; however, when there was considerably less January–March precipitation (18, 25, 45 mm), *B. tectorum* responses were largely either negative or neutral.

*Bromus tectorum* establishment, survival, growth, and reproduction, in areas with limited fall/winter germination, has been found to be highly dependent on spring precipitation (Mack and Pyke 1983; Meyer et al. 2001; Bradford and Lauenroth 2006; Roundy et al. 2007; Concilio et al. 2013; Zelikova et al. 2013). The low fall germination at our site (Authors, personal observation), in combination with its cold temperatures and relatively low winter precipitation, limits *B. tectorum* winter survival and restricts *B. tectorum*’s effective growing season to April–June. This makes precipitation during this period vital for growth of *B. tectorum*. Therefore, unsurprisingly and consistent with our expectations, when we decreased precipitation during the spring–summer growing season, *B. tectorum* responded negatively. These results were consistent with a recent Northern Great Basin study, which found April and May precipitation to be strong drivers of *B. tectorum* and, when reduced, resulted in low *B. tectorum* cover (Boyte et al. 2016).

Reduced precipitation, observed or manipulated, within the sagebrush/cool season perennial grasslands has resulted in decreased native grass cover and abundance (Anderson and Inouye 2001; Heitschmidt et al. 2005). When precipitation was experimentally decreased in conjunction with increased temperatures, total community production (Harte and Shaw 1995) and graminoid growth (Cherwin and Knapp 2012) of two separate Colorado sagebrush steppe sites was reduced. Similarly, it has previously been shown that *P. spicata* is unable to extract water from extremely dry soils and responds negatively to drought conditions, thus it has a limited capacity to respond to the combination of reduced water and increased temperatures (Harris 1967; Cline et al. 1977; Fraser et al. 2009). Thus, it was not surprising that our warming + drying treatment negatively affected the overall cover of the native grass community and the cover and abundance of *P. spicata*.

### Bromus tectorum, climate, and the native grass community

Despite the demonstrated importance of climate, native perennial grass communities are the most important factor affecting *B. tectorum* growth and landscape position (Brummer et al. 2016). Similarly, in situ manipulative climate studies have found that native perennial grass community abundance better explains *B. tectorum* abundance than either temperature (Compagnoni and Adler 2014a) or precipitation (Prevéy and Seastedt 2015). Consistent with these studies and our expectations, the native grass community had a larger suppressive effect on *B. tectorum* abundance than either climate treatment.

*Bromus tectorum* has responded positively to experimentally increased temperatures (Zelikova et al. 2013; Compagnoni and Adler 2014a, b; Blumenthal et al. 2016) and has a shallow diffuse root system, which allows it to maximize use of available water, making it competitive with perennial sagebrush grasses under dry spring/summer conditions (Hull 1963; Harris 1967; Link et al. 1990). In a controlled setting, Hull (1963) found that *B. tectorum* had higher water use efficiency than a common perennial competitor, needing 0.66 as much water to produce 1 g of dry matter. Harris (1967) reported that *B. tectorum* was more competitive for soil water with seedlings of two perennial competitors, including *P. spicata*, because of greater root growth rates during the winter. Finally, a controlled setting experiment demonstrated that warm and dry conditions enhanced *B. tectorum*’s competitiveness with established *P. spicata* individuals (Larson et al. unpublished data). Furthermore, *B. tectorum* cover, as well as warm and dry climate conditions, has been associated with reduced community biodiversity and richness (Ashton et al. 2016; Bansal and Sheley 2016). Therefore, we investigated if *B. tectorum* would be more competitive with the native grass community and would have larger effects on community biodiversity in warm and dry growing season conditions. This did not occur. *Pseudoroegneria spicata*, the native grass community, and the community biodiversity indices demonstrated negative relationships with *B. tectorum*. However, the cold soils and the low late winter/early spring precipitation of our site did not allow for early *B. tectorum* establishment and growth. Thus, our *B. tectorum* populations did not have the established diffuse root systems needed to maximize use of available soil water, which has previously given *B. tectorum* a competitive advantage over native competitors in dry spring/summer conditions. As such, our warming and drying treatment only intensified *B. tectorum* water stress during the growing season and failed to increase its competitiveness with the native grass community and its effects on community biodiversity metrics.

### The effects of fire on the native grass community and *Bromus tectorum*

A decrease in native grass cover the first growing season after a fire is common (Bailey and Anderson 1978; Whisenant and Uresk 1989; West and Yorks 2002; Davies et al. 2012; Reed-dustin et al. 2016). Miller et al. (2013) reported that 86% of the Great Basin fire literature demonstrated a decrease in perennial grass cover the first year after a burn. Therefore, the negative response by the native grass community to the burn treatment is both consistent with the literature and was expected. Factors affecting these responses include slope, aspect, topography, and climate (Reed-dustin et al. 2016). Studies have found that experimentally decreased precipitation and increased temperatures after a burn have decreased native community resilience, resulting in limited post-fire recovery, consistent with significant community shifts away from pre-fire conditions (Enright et al. 2014). Consistent with the literature, the native grass community cover responded to our burn treatment and was sensitive to our post-fire climate conditions. Thus, we conclude that in the first year after a fire our imposed warmer and warmer + drier conditions decreased native community resilience. While an initial negative response to fire is expected by native grass communities, they also respond quickly and are often back to pre-fire conditions within 2–3 years (Baily and Anderson 1978; West and Yorks 2002; Davies et al. 2012). Therefore, conclusions about the long-term resilience to fire of our site under warmer and warmer and drier conditions are limited.

Using experimental warming, warming + drying, and an experimental burn, we addressed the bioclimatic envelope models which have posited that, given adequate winter/spring precipitation, increased summer temperatures and decreased summer precipitation resulting from global climate change will increase *B. tectorum*’s invasiveness and may initiate the positive feedback between fire and *B. tectorum* in sagebrush ecosystems where they have previously been limited by climates with cooler mean temperatures and receive the majority of their precipitation in the summer, such as Montana (Bradley 2009; Taylor et al. 2014; Bradley et al. 2016). However, because of our site’s low late winter/early spring precipitation, the results of our experimental warming and warming + drying treatments had deleterious effects on *B. tectorum* growth and abundance. Furthermore, we only observed a limited positive response by *B. tectorum* to fire, which was not heightened by the experimental warming and drying. Overall, these results add to other studies showing a lack of positive fire-feedback in the cold and wet northern sagebrush biome, including Montana (Taylor et al. 2014). Despite lowered native community resilience to fire in our warming and warming + drying treatments, our short-term results provide three main findings. In the colder northern sagebrush biome that receives more summer than winter precipitation: (1) warmer and drier growing season conditions lower ecosystem resilience to disturbance; but (2) the threat of *B. tectorum* becoming a transformative species as the result of climate warming is low unless there is a shift in seasonal precipitation, where more winter precipitation could facilitate earlier *B. tectorum* establishment and growth; and (3) the effects of climate change may be modified by spring burning, which negatively impacts native grasses more than it does *B. tectorum*, potentially leading to an indirect positive impact of climate on *B. tectorum*.

## Electronic supplementary material

Below is the link to the electronic supplementary material.

Supplementary material 1 (PDF 100 kb)

Supplementary material 2 (PDF 148 kb)

Supplementary material 3 (PDF 149 kb)

Supplementary material 4 (PDF 148 kb)

Supplementary material 5 (PDF 92 kb)

Supplementary material 6 (PDF 164 kb)

Supplementary material 7 (PDF 165 kb)
