# Supplementary material for: A warmer and drier climate in the northern sagebrush biome does not promote cheatgrass invasion or change its response to fire
Source: Oecologia. 2017 Oct 16;185(4):763–74. doi: 10.1007/s00442-017-3976-3 (PMC5681598; doi:10.1007/s00442-017-3976-3)
Supplement: Supplementary file 1 — Supplementary material 1 (PDF 100 kb) [file 442_2017_3976_MOESM1_ESM.pdf]

Online Resource 1 Temperature and precipitation data for Norris climate station (1908-2016). Norris climate data were downloaded from NOAA's National Centers for Environmental Information (http://www.ncdc.noaa.gov/).

		Mean temp. (˚C)	Mean min. temp. (◦C)	Mean max. temp. (◦C)	Total precip. (mm)
Annual	1908-1983	8.10	1.80	14.40	451.10
	1984-2013	9.09	2.82	15.28	424.15
	2014	9.54	3.73	15.33	611.64
	2015	10.81	4.88	16.79	563.36
	2016	10.63	4.78	16.47	512.31
Jan-March	1908-1983	-1.15	-6.24	3.94	63.85
	1984-2013	0.85	-4.17	5.84	50.85
	2014	1.06	-3.48	5.57	128.53
	2015	4.72	-0.56	9.98	46.98
	2016	4.00	-0.71	8.71	78.99
April-June	1908-1983	11.52	4.71	18.32	196.76
	1984-2013	12.24	5.55	18.89	194.90
	2014	12.41	6.11	18.68	217.17
	2015	13.59	7.18	19.98	232.67
	2016	13.94	7.33	20.56	174.75
July-Sept.	1908-1983	18.57	10.45	26.67	113.06
	1984-2013	19.35	11.33	27.34	106.36
	2014	19.29	11.94	26.6	170.69
	2015	20.02	12.39	27.74	137.41
	2016	19.33	11.94	26.74	121.16
Oct.-Dec.	1908-1983	3.33	-1.90	8.55	78.26
	1984-2013	3.53	-1.57	8.60	70.07
	2014	5.59	0.34	10.44	95.25
	2015	4.93	0.50	9.44	146.30
	2016	5.22	0.54	9.87	137.41
