# Supplementary material for: A warmer and drier climate in the northern sagebrush biome does not promote cheatgrass invasion or change its response to fire
Source: Oecologia. 2017 Oct 16;185(4):763–74. doi: 10.1007/s00442-017-3976-3 (PMC5681598; doi:10.1007/s00442-017-3976-3)
Supplement: Supplementary file 3 — Supplementary material 3 (PDF 149 kb) [file 442_2017_3976_MOESM3_ESM.pdf]

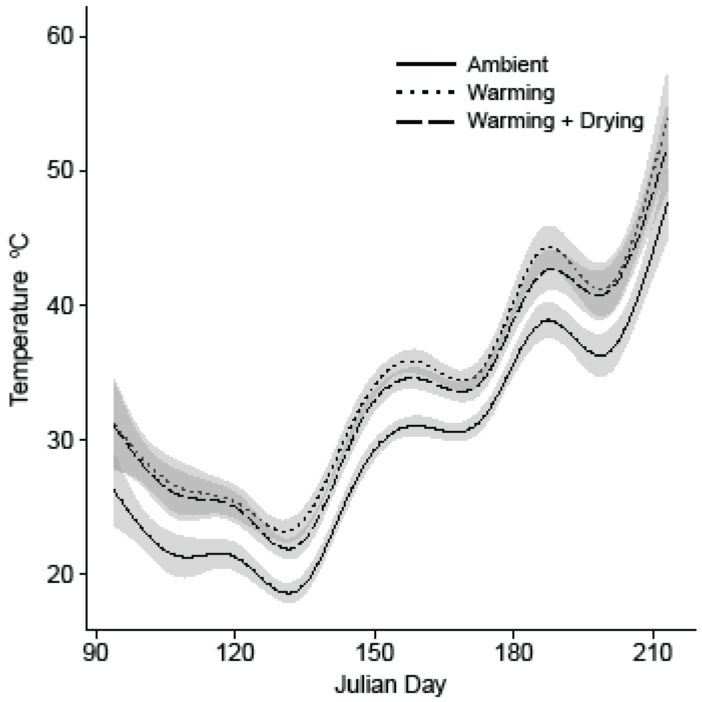

Online Resource 3 Mean maximum temperature data for the ambient (solid line), warming (dotted line), and warming + drying (dashed line) climate treatments (2014 – 2016), with the associated 95% confidence interval.
